# Association between Hepatic Oxidative Stress Related Factors and Activation of Wnt/β-Catenin Signaling in NAFLD-Induced Hepatocellular Carcinoma

**DOI:** 10.3390/cancers14092066

**Published:** 2022-04-20

**Authors:** Kota Hamaguchi, Koji Miyanishi, Takahiro Osuga, Shingo Tanaka, Ryo Ito, Hiroki Sakamoto, Tomohiro Kubo, Hiroyuki Ohnuma, Kazuyuki Murase, Kohichi Takada, Minoru Nagayama, Yasutoshi Kimura, Toru Mizuguchi, Ichiro Takemasa, Junji Kato

**Affiliations:** 1 Department of Medical Oncology, Sapporo Medical University School of Medicine, Sapporo 060-8543, Japan; ko-hamaguchi@sapmed.ac.jp (K.H.); t.osuga@sapmed.ac.jp (T.O.); stanaka@sapmed.ac.jp (S.T.); itoryo@sapmed.ac.jp (R.I.); h.sakamoto@sapmed.ac.jp (H.S.); kubotomo@sapmed.ac.jp (T.K.); ohnuma@sapmed.ac.jp (H.O.); murase@sapmed.ac.jp (K.M.); ktakada@sapmed.ac.jp (K.T.); jkato@sapmed.ac.jp (J.K.); 2 Department of Infection Control and Laboratory Medicine, Sapporo Medical University School of Medicine, Sapporo 060-8543, Japan; 3 Department of Surgery, Surgical Oncology and Science, Sapporo Medical University School of Medicine, Sapporo 060-8543, Japan; mnagayam@sapmed.ac.jp (M.N.); imuray@sapmed.ac.jp (Y.K.); tmizu@sapmed.ac.jp (T.M.); itakemasa@sapmed.ac.jp (I.T.); 4 Postgraduate School of Health Science and Medicine, Sapporo Medical University, Sapporo 060-8543, Japan

**Keywords:** Gd-EOB-DTPA-enhanced MRI, nonalcoholic fatty liver disease, iron overload, hepatocellular carcinoma, serum ferritin, Wnt/β-catenin signaling pathway, immune checkpoint inhibitor

## Abstract

**Simple Summary:**

Activation of the Wnt/β-catenin signaling pathway may reduce the efficacy of immune checkpoint inhibitors, which are first-line therapeutic agents for the treatment of hepatocellular carcinoma (HCC). Following gadoxetate-enhanced MRI, HCC lesions may exhibit equal or higher signal intensities in the hepatobiliary phase than normal tissue. Thus, MRI could be used to monitor the therapeutic effect of antitumor agents. In this study, we investigated the relationship between intrahepatic iron overload markers and oxidative stress and activation of the Wnt/β-catenin signaling pathway. We found that for nonalcoholic fatty liver disease-induced HCC, MRI yielded a sensitivity of 57.2% and a specificity of 100%. Serum ferritin > 77.5 ng/mL had a sensitivity of 85.7% and a specificity of 85.7%. We conclude that serum ferritin levels may further improve the accuracy with which activation of Wnt/β-catenin signaling can be predicted.

**Abstract:**

We investigated the association between iron overload, oxidative stress (8-oxo-7,8-dihydroguanine: 8-oxo-dG scores), Wnt/β-catenin pathway activation (expression of glutamine synthetase: GS), and tumor hyperintensity in the Gd-EOB-DTPA-enhanced MRI hepatobiliary phase (relative enhancement ratio: RER). This was a retrospective analysis of 94 hepatocellular carcinoma (HCC) patients who underwent surgical resection. In HBV-, HCV-, and alcohol-associated HCC, serum ferritin levels in the high and low RER groups were equivalent. In contrast, ferritin levels were elevated in the ‘high RER’ group of patients with nonalcoholic fatty liver disease (NAFLD)-HCC. As predictors of GS positivity, high RER had a sensitivity of 57.2% and a specificity of 100%. High serum ferritin had a sensitivity of 85.7% and a specificity of 85.7%. All cases with serum ferritin ≥275.5 ng/mL and high RER were 8-oxo-dG- and iron staining-positive. Additionally, GS positivity was seen in all cases with “serum ferritin levels above the upper limits or iron staining-positive” and ‘8-oxo-dG high’ cases. Therefore, combining serum ferritin levels with RER may increase the accuracy with which activated Wnt/β-catenin signaling is predicted in NAFLD-HCC. We suggest that 8-oxo-dG accumulates following increased oxidative stress due to hepatic tissue iron deposition; this may activate Wnt/β-catenin signaling and trigger carcinogenesis.

## 1. Introduction

Excess iron leads to oxidative stress and the production of 8-oxo-7,8-dihydroguanine (8-oxo-dG) [1], which induces point mutations in DNA [2]. This process may contribute to liver carcinogenesis [3,4,5]. We have previously shown that some non-alcoholic steatohepatitis (NASH) patients have intrahepatic iron overload and that tumor incidence is higher in patients with general 8-oxo-dG accumulation in the liver [6]. We also generated a murine NASH model in which MUTYH knockout mice (that are unable to repair mutations caused by 8-oxo-dG) are fed a diet high in fat, carbohydrate, and iron; liver carcinogenesis was significantly increased in these mice. We also found that Wnt/β-catenin signaling was activated in the animals [7]. Although increased oxidative stress due to excess iron and Wnt/β-catenin activation is correlated in this mouse model, the mechanistic relationships between these events have not been characterized in humans.

In recent years, combination therapy with an immune checkpoint inhibitor (ICI) and a molecular-targeted drug [8] has become one of the standard treatments for hepatocellular carcinoma (HCC) with distant metastasis. Previously, transcatheter arterial chemoembolization (TACE) was only used in HCC of Barcelona Clinic Liver Cancer (BCLC) staging B, which corresponds to the stage of multiple HCC localized to the liver. Currently, however, systemic drug therapy is the standard treatment alongside TACE [9]. However, there are a considerable number of HCC for which combination therapy with an ICI and a molecular-targeted drug is ineffective. For example, the response rate of a cohort of HCC patients to treatment with atezolizumab and bevacizumab is about 27.3%, and the maximum therapeutic effect observed was stable disease or better in 80.4% of patients and progressive disease (PD) in 19.6% of individuals [8]. Additionally, subgroup analysis of this Phase III trial suggests that current combination therapies are less effective for non-B non-C (NBNC)-HCC [10]. Conventional molecular-targeted drugs should have some therapeutic effect [11,12]. Therefore, prospective stratification of HCC patients into predicted ‘responder’ and ‘non-responder’ groups would facilitate selection of the most appropriate therapeutic agents. This stratification is important for improving both treatment strategy and medical economics. However, there is no established method for identifying such patient groups at present, and a useful predictive biomarker is required. A promising marker is activation of the Wnt/β-catenin signaling pathway [13,14]. Tumors in which the Wnt/β-catenin pathway is activated are often considered to be so-called ‘cold tumors’, as they have less infiltration of T lymphocytes, which may reduce the efficacy of ICIs [15,16,17]. When measured with Gd-EOB-DTPA-enhanced MRI, HCCs with activated Wnt/β-catenin signaling have hepatobiliary phase signals equal to or higher than those of background liver. Thus, this difference in contrast effect may be an indirect marker for predicting the effects of ICIs in Wnt-driven and Wnt-off cancers [18].

Here, we investigated the association between serum ferritin levels, which reflects iron overload, non-cancerous 8-oxo-dG, which reflects oxidative DNA damage, expression of glutamine synthetase (GS) in the cancerous region, which reflects Wnt/β-catenin signaling pathway activation [19,20], and the hyperintensity of tumors following Gd-EOB-DTPA-enhanced MRI. We then determined whether these parameters could be used to predict the therapeutic effect of ICIs.

## 2. Materials and Methods

### 2.1. Patients and Tissue Samples

We included 94 pathologically diagnosed HCC patients who were treated at the Division of Medical Oncology, Sapporo Medical University Hospital, and received hepatectomy at the Division of Surgery, Surgical Oncology and Science, Sapporo Medical University Hospital. We performed a retrospective analysis using data from the electronic medical record and from the analysis of specimens extracted by surgical operation.

### 2.2. Definitions and Criteria

We defined patients who were positive for HBsAg at the time of surgery as HBV-HCC, patients who were positive for HCV antibody as HCV-HCC, and patients who were negative for both as NBNC-HCC. The NBNC-HCC patients were divided into subgroups as follows. Patients with a history of polydipsia (60 g ethanol/day or more for males, and 40 g ethanol/day or more for females) were defined as having alcohol liver disease (ALD)-HCC, and patients without alcohol polydipsia were defined as having nonALD-NBNC-HCC. Among nonALD-NBNC-HCC, we defined NAFLD-HCC as patients who had no history of drinking or who drank very little (less than 30 g ethanol/day for males, less than 20 g ethanol/day for females) and had pathological fat deposition in non-cancerous areas of excised tissue or fatty liver changes on diagnostic imaging.

### 2.3. Assessment of Tissue Iron Accumulation

To evaluate iron deposition, serum ferritin levels and iron staining of tissues were quantified. The serum ferritin levels were analyzed using data acquired within the 2 months immediately before surgical resection. For iron staining, Berlin blue staining was performed using potassium hexacyanoferrate(II) trihydrate (FUJIFILM Wako Pure Chemical Corporation, Osaka, Japan). The degree of iron deposition in liver tissue was assessed by modifying the criteria previously published by Rowe et al. [21]. Briefly, Grade 0 was assigned in the case that granules are absent or almost indistinguishable at 400× magnification; Grade 1 if distinguishable at 400× magnification; Grade 2 if discernible at 200× magnification; Grade 3 if granules could be seen at 50× magnification; and Grade 4 if granules were seen with the naked eye. In this study, grade 0 was evaluated as negative and grade 1 or higher was evaluated as positive.

### 2.4. Immunohistochemical Determination of Glutamine Synthetase

Glutamine synthetase (GS) immunohistochemical staining was performed to evaluate activation of the Wnt/β-Catenin signaling pathway. Immunohistochemical staining used tissue sections fixed in formalin and embedded in paraffin. The slides were deparaffinized and incubated with BOND Novocastra Epitope Retrieval Solution pH 6 (Leica Biosystems, Nussloch, Germany) for 20 min at 100 °C for Heat-Induced Epitope Retrieval. After incubation with a 3% hydrogen peroxide solution for 5 min to inactivate the endogenous peroxidase, samples were treated with a blocking agent (Block Ace; KAC Co., Ltd., Kyoto, Japan) for 10 min to prevent a non-specific reaction. Samples were reacted with the anti-Glutamine synthetase antibody (Merck Millipore, Billerica, MA, USA, 1:500 dilution) as the primary antibody for 15 min. After that, the reaction with secondary antibody and color development was performed with BOND Polymer Refine Detection (Leica Biosystems, Nussloch, Germany). The samples were reacted with the secondary antibody solution in the kit for 8 min and then reacted with 3, 3-diaminobenzidine solution in the kit for 10 min for color development. The samples were then reacted with hematoxylin in the kit for 3 min to stain nuclei. As previously reported [22], GS was evaluated as positive when almost all tumor cells were homogenously stained or when more than 50% of tumor cells were heterogeneously stained.

### 2.5. Semi-Quantitative Assessment of Hepatic 8-oxo-dG

To make a semi-quantitative evaluation of 8-oxo-dG in the liver, immunohistochemical staining with 8-oxo-dG antibody was performed as outlined above using an anti-8-OHdG primary monoclonal antibody (Japan Institute for the Control of Aging, Fukuroi, Japan, 1:100 dilution). The intensity of 8-oxo-dG immunostaining in sections was assessed by modifying the criteria previously published by Gong et al. [23]. Briefly, quantitative evaluation of positive cells was performed as follows: positive cells ≤25%, 0; positive cells 26–50%, 1; positive cells 51–75%, 2; positive cells >75%, 3. The staining intensity was evaluated as follows: no staining, 0; light brown, 1; brown, 2; and dark brown, 3. Overall scores were obtained by multiplying the positive cell score and the staining intensity score. The overall score for three periportal regions and three perivenous regions was obtained, and the average of all the scores was taken as the 8-oxo-dG score.

### 2.6. Assessment of the Hepatobiliary Phase of Gd-EOB-DTPA-Enhanced MRI

To evaluate activation of the Wnt/β-Catenin pathway by the Gd-EOB-DTPA-enhanced MRI hepatobiliary phase, we used the relative enhancement ratio (RER) [18,24]. The calculation method of RER is as shown below. To measure the signal intensity (SI) of tumor nodules and background liver parenchyma of HCC, regions of interest (ROIs) were determined. The signal intensity (SI) of tumor nodules was set using a circular ROI that included as much of the tumor as possible in the cross-section where the tumor diameter is maximized in the horizontal section. In addition, we set three ROIs in the background liver parenchyma, avoiding the portal vein, major branches of the hepatic veins, and artifacts. In each phase, the mean of the three ROIs was recorded as the parenchyma SI. In principle, the ROI was set to at least 40 mm^2^. However, ROIs were set as large as possible for tumors with small diameters. The SI of the tumor nodule was named SInod, the SI of the background liver parenchyma was named SIpar, and the relative intensity ratio (RIR) = SInod/SIpar was calculated. The RIR in the hepatobiliary phase was named RIRHBP, the precontrast RIR was named RIRpre, and the relative enhancement ratio (RER) = RIRHBP/RIRpre was calculated. Gd-EOB-DTPA-enhanced images were analyzed by two hepatologists (KH and TO) using the data immediately before surgical resection (within two months at the longest). Consistent with previous reports [18,24], we defined an RER ≥ 0.9 as positive.

### 2.7. Statistical Analysis

All values are expressed as median (range). Two groups were compared by using the Fisher exact test for categorical data and the Wilcoxon Mann–Whitney U test for quantitative data. ROC curves were used to determine the cutoff values of biomarkers. All statistical analyses were performed using Easy R (EZR) version 1.42 software (Saitama Medical Center, Jichi Medical University, Saitama, Japan) [25] or GraphPad Prism 9 version 9.2.0 software (GraphPad Software, LCC, CA, USA).

## 3. Results

### 3.1. Clinical Characteristics of HCC Patients

A total of 94 consecutive patients (65 males and 29 females) with HCC who underwent surgical resection at our hospital between 1 January 2011 and 31 August 2021 were investigated in this study. The median patient age was 71 years (47–88 years). Serum ferritin was evaluated preoperatively in 86 cases with a median of 143.5 ng/mL (1.5–2674). Preoperative Gd-EOB-DTPA-enhanced MRI was evaluated in 88 cases. Regarding the etiology, there were 27 cases of HBV-HCC, 29 cases of HCV-HCC, 13 cases of ALC-HCC, 14 cases of NAFLD-HCC, and 11 cases of others. Other baseline characteristics are shown in Supplementary Table S1.

### 3.2. Relationship between Serum Ferritin Levels and Gd-EOB-DTPA-Enhanced MRI Hepatobiliary Phase Findings by HCC Etiology

**Figure 1:**
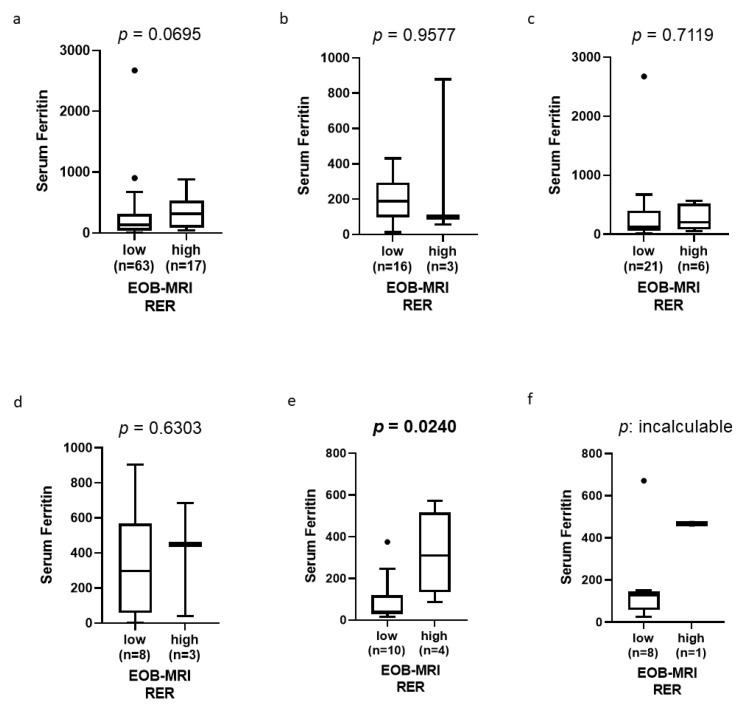
The relationships between RER, which has been associated with activation of Wnt/β-Catenin signaling, and serum ferritin levels. (**a**) All etiology, (**b**) HBV-HCC, (**c**) HCV-HCC, (**d**) ALD-HCC, (**e**) NAFLD-HCC, (**f**) others.

In the group of patients who had been evaluated for serum ferritin and Gd-EOB-DTPA-enhanced MRI before surgery, the relationship between serum ferritin levels and Gd-EOB-DTPA-enhanced MRI RER was examined separately by etiology. In HBV-HCC, HCV-HCC, and ALC-HCC, there was no difference in serum ferritin levels between high and low RER cases. On the other hand, in NAFLD-HCC, serum ferritin levels were significantly higher in high RER cases than in low RER cases (median 309.1 ng/mL vs. 41.2 ng/mL, *p* = 0.0240; Figure 1). ROC curve analysis revealed that when the cutoff value of serum ferritin was 86.1 ng/mL, the sensitivity was 100% and the specificity was 80.0% for predicting high RER in NAFLD-HCC (Supplementary Figure S2a). In the analysis of nonALD-NBNC-HCC, the serum ferritin levels were significantly higher in high RER cases (median 342.7 ng/mL vs. 74.3 ng/mL, *p* = 0.0193; Supplementary Figure S1).

### 3.3. Tissue Iron Accumulation in Non-Cancerous Areas and RER in Cancerous Areas

**Figure 2:**
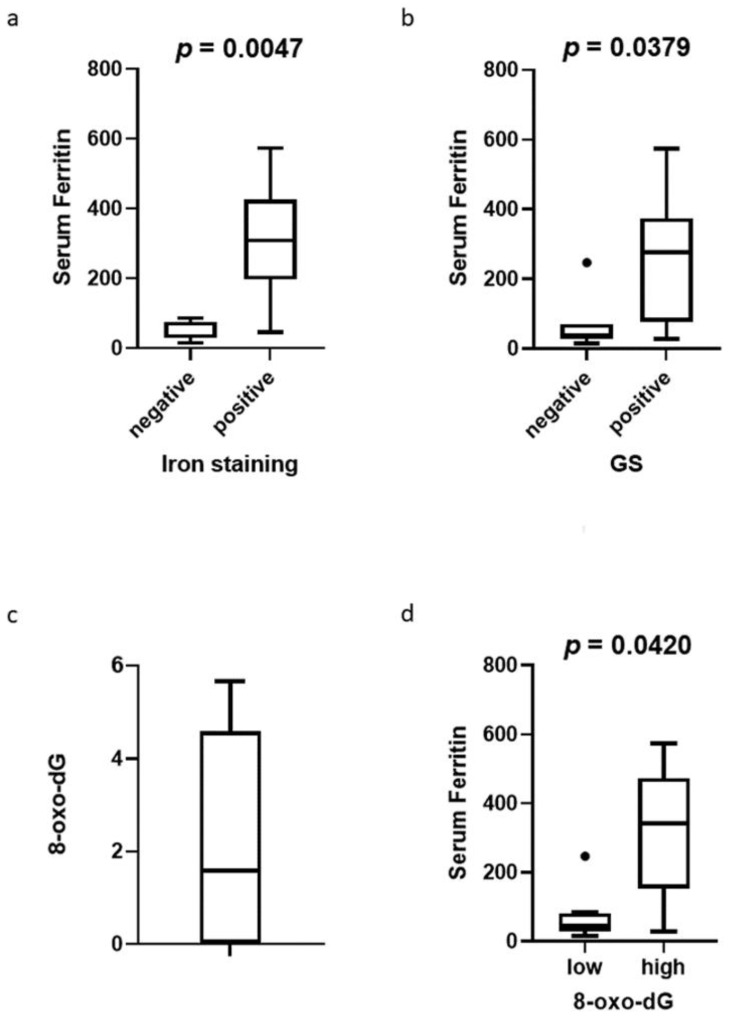
The relationships between immunohistochemical staining and serum ferritin levels. (**a**) Serum ferritin levels in iron staining-negative and -positive cases. (**b**) Serum ferritin levels in GS-negative and -positive cases. (**c**) 8-oxo-dG scores of non-cancerous areas in the liver in all NAFLD-HCC cases. (**d**) Serum ferritin levels compared between 8-oxo-dG low cases and high cases.

In NAFLD-HCC, serum ferritin levels were significantly higher in high RER cases than low RER cases. Therefore, the relationship between tissue iron deposition in the background liver and RER was analyzed in 14 cases of NAFLD-HCC. Six cases were iron staining positive and eight were negative. Serum ferritin levels were significantly higher in iron staining-positive cases (median 309.1ng/mL vs. 35.3ng/mL, *p* = 0.0047; Figure 2a). This result suggests that hepatic tissue iron deposition is reflected in serum ferritin levels in this particular patient group. We also investigated GS staining in cancerous areas, which has been reported to reflect activation of the Wnt/β-Catenin signaling pathway [20]. RER ≥ 0.9 was predicted to be GS-positive, with a sensitivity of 57.2% and a specificity of 100%. GS-positive cases had significantly higher serum ferritin levels than negative cases (median 275.5 ng/mL vs. 37.6 ng/mL, *p* = 0.0379; Figure 2b). ROC curve analysis showed that when the cutoff value of serum ferritin was 77.5 ng/mL, the sensitivity was 85.7% and the specificity was 85.7% for predicting GS positivity in NAFLD-HCC (Supplementary Figure S2b). Thus, GS-positive cases in NAFLD-HCC could be predicted with high accuracy by serum ferritin levels.

### 3.4. Relationship between 8-oxo-dG in Non-Cancerous Areas, Iron Deposition, and GS Staining

In 14 cases of NAFLD-HCC, the relationship between 8-oxo-dG in the non-cancerous areas, iron deposition, and GS staining was evaluated. The median 8-oxo-dG score was 1.6 (0–5.6; Figure 2c). Since 8-oxo-dG is increased by oxidative stress due to iron deposition in liver tissue [6,7], the cutoff value of the 8-oxo-dG score for predicting the positivity of iron staining in the non-cancerous areas was examined using a ROC curve. With a cutoff 8-oxo-dG score of 4.5, the sensitivity was 66.7% and the specificity was 87.5% for predicting iron staining positive NAFLD-HCC cases (Supplementary Figure S2c). The population was divided into 8-oxo-dG-high cases or 8-oxo-dG-low cases using this cutoff value and analysis was performed. 8-oxo-dG-high cases had significantly higher serum ferritin levels than low cases (median 342.7ng/mL vs. 44.8ng/mL, *p* = 0.042; Figure 2d). Moreover, when using the cutoff value mentioned above, high 8-oxo-dG predicted GS positivity with a sensitivity of 57.1% and a specificity of 77.8%. Iron staining was able to predict GS positivity with a sensitivity and specificity of 57.1% and 71.4%, respectively. There were four cases in which serum ferritin levels were above the upper limit of the reference range (250 ng/mL for male, 120 ng/mL for female) and 8-oxo-dG scores were 4.5 or higher. These four cases were also iron staining-positive in non-cancerous areas of the liver and GS positive in cancerous areas.

These results suggest that in NAFLD-HCC, activation of the Wnt/β-Catenin signaling pathway may occur in patients with iron deposition and high 8-oxo-dG levels in non-cancerous areas in the liver.

In the NAFLD-HCC group, we also compared the GS-positive and -negative sub-groups with respect to the following characteristics: differences in age (*p* = 0.6367), sex (*p* > 0.9999), hemoglobin (*p* = 0.9747), platelets (*p* = 0.2284), total bilirubin (*p* = 0.0173), albumin (*p* = 0.5045), aspartate aminotransferase (*p* = 0.8808), alanine aminotransferase (*p* = 0.1002), prothrombin time (0.9497), alpha-fetoprotein (*p* = 0.5589), protein induced by vitamin K absence or antagonist-II (*p* = 0.5511), Child–Pugh class (*p* = 0.4286), Barcelona Clinic Liver Cancer stage (*p* > 0.9999), and 8th edition of the Union for International Cancer Control stage (*p* = 0.4069). Total bilirubin was significantly higher in the GS positive group, although only in a small number of patients, and may be related to the increased production of glutamine by GS, since it has been reported that glutamine promotes bile secretion [26]. No differences were observed in other parameters.

### 3.5. Examination of Cases with Discrepancies between GS and Gd-EOB-DTPA-Enhanced MRI Hepatobiliary Phase Results

**Figure 3:**
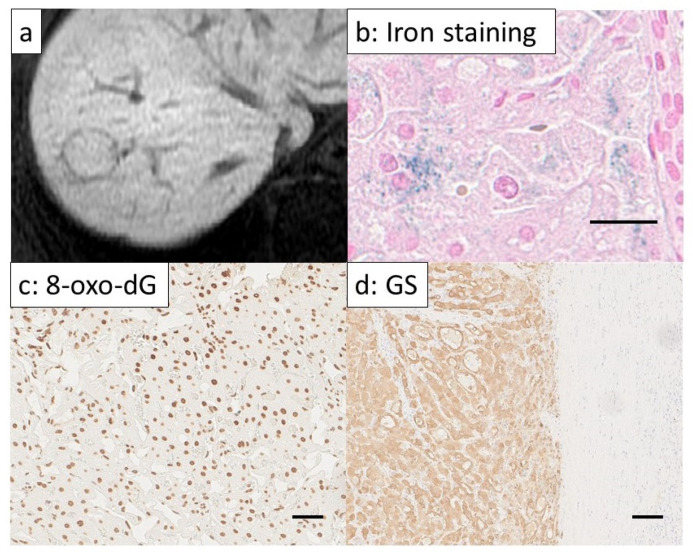
Gd-EOB-DTPA-enhanced MRI image and immunohistochemical staining of hepatocellular carcinoma cases with high serum ferritin level and showing a high signal in the hepatobiliary phase of Gd-EOB-DTPA-enhanced MRI. (**a**) Gd-EOB-DTPA-enhanced MRI (hepatobiliary phase). The tumor showed a high signal. RER = 1.01. (**b**) Iron staining (Berline blue staining) of a non-cancerous area in the liver (×800). Scale bar = 15 μm. Iron deposits were found in the liver tissue of the non-cancerous area. (**c**) Strong 8-oxo-dG staining of non-cancerous area in the liver (×400). Scale bar = 50 μm. (**d**) GS staining at the border between cancerous and non-cancerous areas (×200). Scale bar = 25 μm. It is likely that Wnt/β-Catenin signaling had been activated in this case because the tumor tissue located on the left side of the image was strongly stained.

A previous report showed that an RER ≥ 0.9 for prediction of Wnt/β-Catenin pathway activation had a sensitivity of 78.9% and a specificity of 81.7% [24], suggesting that misjudgment had occurred in some cases. We also found that the sensitivity of RER was as low as 57.2% in our current study. We examined whether sensitivity could be increased by combining with serum ferritin levels in addition to RER. Using a ferritin level of 77.5 ng/mL as a cutoff (the same threshold used for the prediction of GS positivity), high serum ferritin levels or RER > 0.9 predicted GS positivity with a sensitivity of 86% and a specificity of 86%. In the case of NAFLD-HCC, we suggest that combining serum ferritin levels with RER, will lead to a more accurate prediction of Wnt/β-catenin pathway activation. In addition, three cases of serum ferritin levels of 275.5 ng/mL or more with RERs > 0.9 were 8-oxo-dG positive and iron staining positive. In these cases, increasing oxidative stress due to hepatic tissue iron deposition led to the accumulation of 8-oxo-dG, which may have caused activation of the Wnt/β-catenin signaling pathway and carcinogenesis by inducing point mutations in DNA (a representative Gd-EOB-DTPA-enhanced image and immunohistochemical staining of a typical case are presented in Figure 3).

## 4. Discussion

The IMbrave150 study involved a phase III trial comparing the efficacy of sorafenib to the efficacy of combined therapy with the ICI, atezolizumab, and bevacizumab, a VEGF inhibitor. In a subgroup of this study, patients with NBNC-HCC had a median overall survival of 17.0 months for atezolizumab and bevacizumab combination therapy and 18.1 months for sorafenib [10]. In HCC caused by viral hepatitis, atezolizumab and bevacizumab combination therapy had a better therapeutic effect than sorafenib, giving rise to the opinion that ICIs are less effective in NBNC-HCC. However, in the IMbrave150 study, progression-free survival and response rate were better with atezolizumab plus bevacizumab therapy, and the effect of ICIs on NBNC-HCC was controversial. A comparison of HCC patients with etiology limited to NAFLD (a subtype of NBNC-HCC) with those with HCC due to other etiologies found that patients with NAFLD-HCC who received anti-PD-1 or anti-PD-L1 treatment had shorter overall survival compared to patients of other etiologies [27]. ICIs are, therefore, considered ineffective in patients whose HCC is caused by NAFLD. This finding may be explained by observations from recent reports. It has been reported that, in NASH-HCC, absolute numbers of a special CD8+ T cell called a CXCR6+ CD8+ T cell increase by a peculiar mechanism. These T cells may have reduced immune surveillance function, unlike T cells that have cellular cytotoxicity specifically for cancer antigens [28]. CXCR6+ CD8+ T cells induce cell death and cause hepatocyte necrosis and fibrosis in non-cancerous liver tissue [28]. In NASH-HCC mice, PD-1 antibody therapy increased CD8+ T cells in the tumor but did not lead to tumor regression. In addition, when administered to tumor-free NASH mice, the incidence of NASH-HCC and the number and size of tumor nodules increased. Furthermore, administration of anti-PD-1 antibody increased CD8+ PD-1+ CXCR6+ T cells in the tumor. It has been suggested that these T cells induce NASH-HCC rather than activating or performing immune surveillance [27]. Thus, ICI treatment is virtually ineffective in pure NASH-HCC; in addition to the mechanisms outlined above, we suggest that other biological processes, such as activation of Wnt/β-catenin signaling, are likely to contribute to the refractory nature of this disease.

Activation of the Wnt/β-catenin signaling pathway is thought to reduce ICI efficacy. This is because the β-catenin mutation suppresses the intratumoral infiltration of CD8+ T cells whose antitumor effect is activated by ICI [29]. In HCC, β-catenin (CTNNB1) mutation is the third most frequent alteration after TERT promoter mutation and TP53 mutation, and may be present in between 20 to 40% of all cases [30,31,32]. In fact, patients without β-catenin mutation had a disease progression rate of 29% with ICI alone, while patients with β-catenin mutation had a disease progression rate of 100% [14]. In this study, we showed that serum ferritin levels may predict activation of the Wnt/β-catenin signaling pathway to some extent in NAFLD-HCC cases.

Results from Gd-EOB-DTPA-enhanced MRI RER and those related to Wnt/β-catenin pathway activation do not always match [24]. In particular, the use of RER as an index for predicting the effect of ICI is associated with low sensitivity [18]. Here, we showed that the detection sensitivity of Wnt/β-catenin pathway activation cases may be improved by considering intrahepatic iron deposition by serum ferritin levels.

We found that oxidative DNA damage in the background liver of patients with NASH-induced HCC was significantly higher than in the liver of patients whose HCC was associated with other etiologies, such as HBV, HCV, and alcoholic liver disease [6]. In our previous study using a mouse model of NASH, we observed that intrahepatic 8-oxo-dG was higher in NASH mice compared to control mice, and that oxidative stress induced by iron accumulation was a robust promoter of hepatic carcinogenesis. Furthermore, microarray analysis revealed significant activation of Wnt/β-catenin signaling in NASH model mice with HCC, suggesting that the Wnt pathway may be the target for HCC that occurs against the background of excess 8-oxo-dG in the liver [7]. As it was reported that the etiology is not the primary determinant of the mutation spectrum in HCC [30], oxidative DNA damage does not always cause gene mutation. However, based on the result of this study, we suggest that NAFLD may lead to carcinogenesis by increasing oxidative stress due to iron deposition in liver tissue; this would lead to the accumulation of 8-oxo-dG and trigger point mutations in DNA, which in turn would activate Wnt/β-catenin signaling. The limitations of this study are that it is a retrospective analysis and that it is a study of a relatively small number of cases. However, there has been a recent increase in NAFLD-HCC and ICIs have become the first-line treatment for HCC. Therefore, this new finding from the perspective of iron metabolism is important, as it may suggest novel treatment options for NAFLD-HCC. In the future, we plan to search for specific genetic alterations in the Wnt/β-catenin signaling pathway.

## 5. Conclusions

In patients with NAFLD-HCC, we suggest that combining serum ferritin levels with hyperintensity findings in the Gd-EOB-DTPA-enhanced MRI hepatobiliary phase may be beneficial in picking up cases in which the Wnt/β-catenin signaling pathway is active. Some patients with NAFLD-HCC may have carcinogenesis due to iron-driven, 8-oxo-dG-mediated activation of Wnt/β-catenin signaling. This may be one of the reasons why NAFLD-HCC is more resistant to ICIs.

## Data Availability

The datasets generated during and/or analyzed during the current study are included in this article. Further enquiries can be directed to the corresponding author on reasonable request.

## Supplementary Materials

The following supporting information can be downloaded at: https://www.mdpi.com/article/10.3390/cancers14092066/s1, Supplementary Figure S1: The relationships between RER and serum ferritin levels in nonALD-NBNC-HCC; Supplementary Figure S2a: ROC curve of serum ferritin to high RER; Supplementary Figure S2b: ROC curve of serum ferritin to GS positivity; Supplementary Figure S2c: ROC curve of 8-oxo-dG to iron staining positive; Supplementary Table S1: Baseline characteristics of 94 patients with HCC.
