# Supplementary figures and images for: Association between Hepatic Oxidative Stress Related Factors and Activation of Wnt/β-Catenin Signaling in NAFLD-Induced Hepatocellular Carcinoma
Source: Cancers (Basel). 2022 Apr 20;14(9):2066. doi: 10.3390/cancers14092066 (PMC9102393; doi:10.3390/cancers14092066)

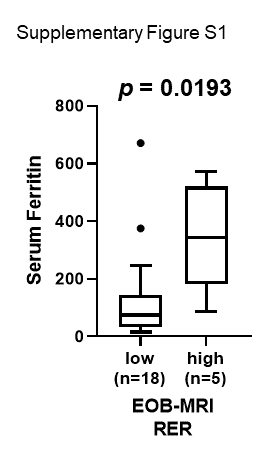

Supplement: Supplementary file 1 [file cancers-14-02066-s001.zip › cancers-1682248-supplementary Figure S1.png]

# Supplementary Figure S2

a

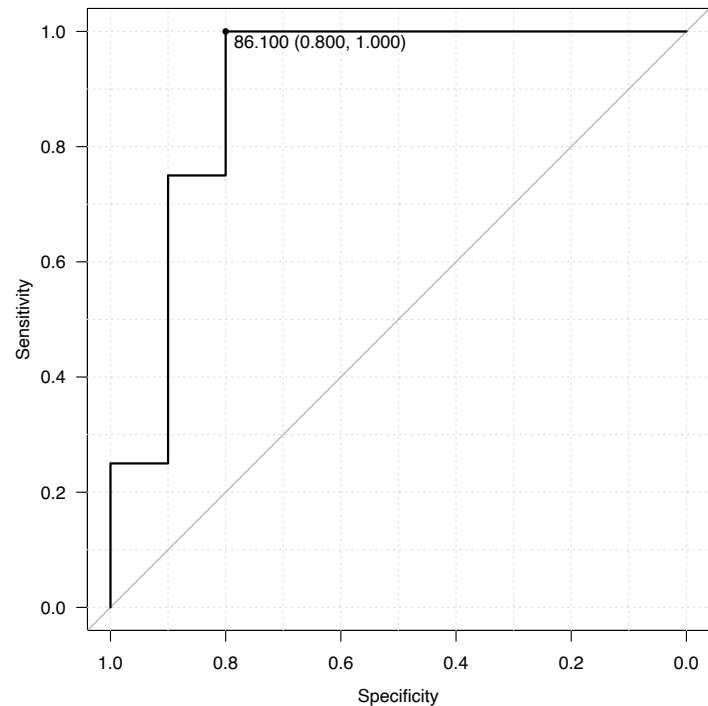

b

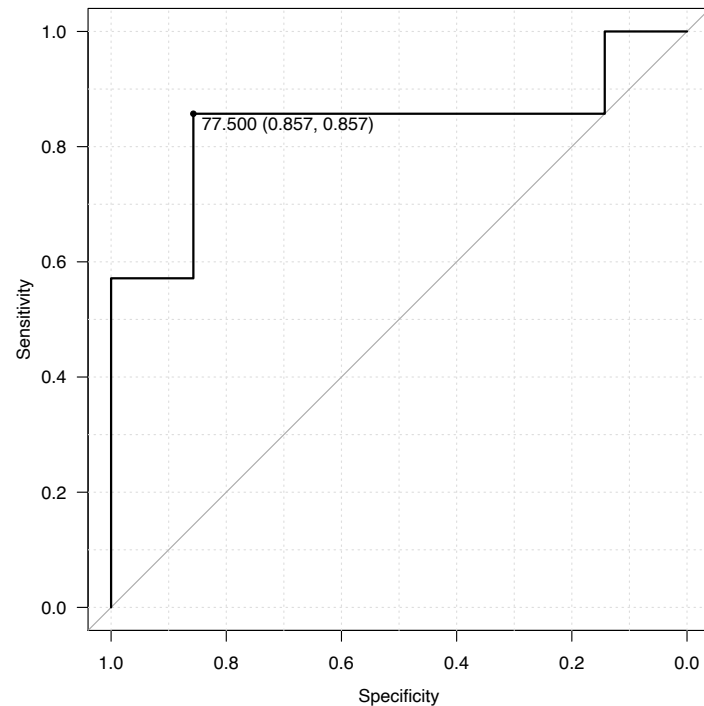

c

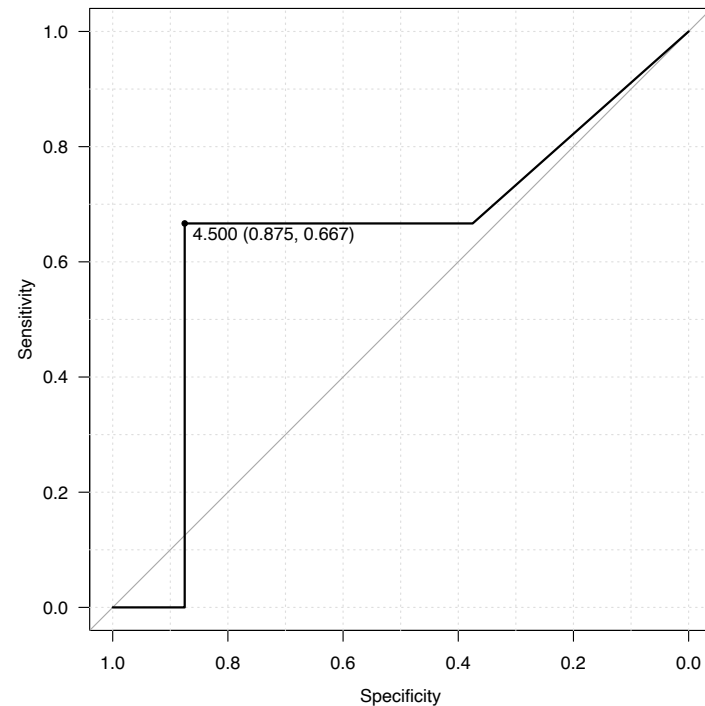

Supplement: Supplementary file 1 [file cancers-14-02066-s001.zip › cancers-1682248-supplementary Figure S2.pdf]
